# A comprehensive analysis of the fatal toxic effects associated with CD19 CAR-T cell therapy

**DOI:** 10.18632/aging.104058

**Published:** 2020-09-24

**Authors:** Changjing Cai, Diya Tang, Ying Han, Edward Shen, Omar Abdihamid, Cao Guo, Hong Shen, Shan Zeng

**Affiliations:** 1 Department of Oncology, Xiangya Hospital, Central South University, Changsha 410008, Hunan, China; 2 Key Laboratory for Molecular Radiation Oncology of Hunan Province, Xiangya Hospital, Central South University, Changsha 410008, Hunan, China; 3 Department of Life Science, McMaster University, Hamilton, ON L8S 4L8, Canada; 4 National Clinical Research Center for Geriatric Disorders, Xiangya Hospital, Central South University, Changsha 410008, Hunan, P.R. China

**Keywords:** CD19 CAR-T cell therapy, immunotherapy, fatal toxic effects, hematological malignancies

## Abstract

To determine the incidence, spectrum, timing, and clinical features of CD19 Chimeric antigen receptor (CAR-T) cell therapy-associated fatal toxic effects. We initiated a comprehensive analysis. First, we retrospectively queried the real-world data from a World Health Organization (WHO) pharmacovigilance database (Vigilyze). Furthermore, we performed a meta-analysis of published trials of CD19 CAR-T cell therapy. From screening the WHO database, we identified 1200 patients: 499 received Kymriah therapy, and 701 received Yescarta therapy. We compared the adverse reactions of the two drugs. We evaluated all published clinical trials, comprising 19 trials and 890 patients. Our meta-analysis showed that the incidence of fatal toxic effects associated with death was 5.4%. Infections and infestations appeared to present the highest risk of death. The toxic effect specific median time to death was 30, 30, and 68 days for total, cytokine release syndrome (CRS), and nervous system disorders (NSD), respectively. We observed a high mortality rate for some toxic effects and an early onset of death with varied causes, indicating the need for clinicians to pay more attention to the monitoring and treatment of these fatal toxic effects when using CD19 CAR-T cell therapy, especially for infections and infestations.

## INTRODUCTION

Chimeric antigen receptor T cell (CAR-T) therapy was first proposed in 1989 [1]. After several decades of innovation, with the approval of Kymriah and Yescarta by the FDA in 2017, CAR-T cell therapy has become one of the important treatments for tumors, especially hematological malignancies [2–5].

With the approval of CAR-T products and the frequent use of CAR-T cell therapy, the toxic effects of treatment have begun to manifest. CAR-T cell therapy can result in the rapid death of cancer cells, which will cause a variety of immune factors to be released, including IL-6, IL-1, IL-12, TNF-α, IFN-α, GM-CSF and so on. The excessive activation of immune factors will trigger severe toxic reactions, such as CRS and nervous system disorders (NSD), and potential death. [6–8].

However, due to the dispersibility of patients using CAR-T cell therapy and the lack of large-scale clinical studies, the types and rate of fatal toxic effects are not yet clear. With increasing uptake in clinical application, the number of deaths caused by the fatal toxicities associated with CD19 CAR-T cell therapy is gradually increasing. Therefore, the analysis of the landscape of fatal toxicity associated with CD19 CAR-T cell therapy is extremely significant.

Currently, the approved CAR-T products are mainly in anti-CD19. Therefore, we selected CD19 CAR-T cell therapy as the study subject and summarized the toxicity reported by all published CD19 CAR-T related clinical trials. We also analyzed the toxicity data of Kymriah and Yescarta in the WHO database (VigiBase-Vigilyze) [9].

We systematically analyzed the probability, timing, and types of fatal toxic effects, and we hope that this study will provide a guideline for clinicians and reduce patients’ mortality caused by the fatal toxic effects associated with CD19 CAR-T cell therapy.

## RESULTS

### Global pharmacovigilance analysis

To evaluate the spectrum of CD19 CAR-T cell-related toxic effects, we reviewed the global adverse drug reaction database (Vigilyze-VigiBase). From screening the database, we identified 1200 patients: 499 received Kymriah therapy, and 701 received Yescarta therapy (Table 1).

**Table 1:** Spectrum of adverse events in vigilyze.

**Variable**	**No. (%)**		**P**
	**Kymriah (n=499)**	**Yescarta (n=701)**	
Type of ADR			
Immune system disorders	261 (52)	433 (62)	0.001
Cytokine release syndrome (CRS)	237 (47)	426 (61)	< 0.001
Nervous system disorders	153 (31)	422 (60)	
General disorders and administration site conditions	259 (52)	286 (41)	
Blood and lymphatic system disorders	150 (30)	137 (20)	
Cardiac disorders	58 (12)	119 (17)	0.1
Psychiatric disorders	52 (10)	109 (16)	0.01
Vascular disorders	99 (20)	104 (15)	0.024
Infections and infestations	112 (22)	99 (14)	< 0.001
Investigations	173 (35)	92 (13)	
Respiratory, thoracic and mediastinal disorders	89 (18)	83 (12)	0.004
Gastrointestinal disorders	64 (13)	72 (10)	0.196
Benign Neoplasms, Malignant Neoplasm and unspecified (incl cysts and polyps)	120 (24)	49 (7)	< 0.001
Renal and urinary disorders	39 (8)	39 (6)	0.124
Metabolism and nutrition disorders	49 (10)	29 (4)	< 0.001
Injury, poisoning and procedural complications	21 (4)	26 (4)	0.654
Musculoskeletal and connective tissue disorders	26 (5)	25 (4)	0.191
Skin and subcutaneous tissue disorders	23 (5)	15 (2)	0.019
Surgical and medical procedures	0	15 (2)	0.031
Hepatobiliary disorders	15 (3)	11 (2)	0.108
Eye disorders	17 (3.4)	8 (1)	0.12
Endocrine disorders	3 (0.6)	7 (0.9)	0.536
Social circumstances	1 (0.2)	5 (0.7)	0.41
Congenital, familial and genetic disorders	1 (0.2)	3 (0.4)	0.645
Product issues	14 (3)	3 (0.4)	0.001
Reproductive system and breast disorders	2 (0.4)	1 (0.1)	0.574
Ear and labyrinth disorders	3 (0.6)	0	0.072
Age group			
28 days to 23 months	7	—	
2 - 11 years	106	—	
12 - 17 years	85	1	
18 - 44 years	95	80	
45 - 64 years	43	245	
65 - 74 years	29	141	
≥ 75 years	7	23	
Unknown	127	211	
ADR reports per year			
2019 (up to August 2019)	322	585	
2018	162	116	
2017	8	—	
2016	5	—	
2015	2	—	
Geographical distribution			
Americas	407	623	
Europe	82	78	
Oceania	10	—	

The types of fatal toxic effects associated with CD19 CAR-T cell therapy depend on the regimens (Table 1, Supplementary Table 1 in the Supplement). With Kymriah therapy, immune system disorders, general and administration site conditions, and cytokine release syndrome (CRS) was highly predominant (261 [52%], 259 [52%], and 237 [47%], respectively). For Yescarta, patients had a wide variety of fatal toxic effects, including immune system disorders (433 [62%]), cytokine release syndrome (CRS) (426 [61%]), nervous system disorders (422 [60%]), general and administration site conditions (286 [41%]), and blood and lymphatic system disorders (137 [20%]).

In addition, we compared the adverse reactions of the two drugs. The incidence of general and administration site conditions; blood and lymphatic system disorders; vascular disorders; infections and infestations; investigations; respiratory, thoracic and mediastinal disorders; neoplasms, including benign, malignant and unspecified (including cysts and polyps); metabolism and nutrition disorders; skin and subcutaneous tissue disorders; and product-related issues were higher in Kymriah therapy than in Yescarta (*P* < 0.05). Conversely, immune system disorders, CRS, NSD, psychiatric disorders, and surgical and medical procedures were lower in Kymriah therapy than in Yescarta (*P* < 0.05). There was no significant difference in cardiac disorders; congenital, familial and genetic disorders; endocrine disorders; or other disorders (*P* > 0.05) (Table 1).

### Meta-analysis

Although the WHO databases are useful for defining the clinical characteristics and spectrum of toxic effects, these databases are unable to conclusively establish their frequency. To determine the frequency of fatal toxic effects, we evaluated all published clinical trials of CD19 CAR-T cell therapy, comprising 19 trials and 890 patients.

To provide additional validation regarding the spectrum of CD19 CAR-T cell-related fatal toxic effects, we assessed the types of fatal toxic effects among the 505 published events (Supplementary Table 2 in the Supplement). CRS was the most frequent cause of death with CD19 CAR-T cell therapy (10 of 33 CD19 CAR-T-related deaths). Nervous system disorders occurred in 6 (18.18%) patients, infections, and infestations in 4 (12.12%), and blood and lymphatic system disorders in 3 (9.09%). Of 7 Kymriah-associated deaths, 2 resulted from blood and lymphatic system disorders, 1 from a nervous system disorder, and 1 from infection and infestation. Of 9 Yescarta-associated deaths, 2 resulted from CRS; 2 from respiratory, thoracic, and mediastinal disorders; and 2 from unknown causes (Table 2). The incidence of fatal toxic effects mortality was not different between Kymriah and Yescarta in all types (*P* > 0.05, Table 2).

**Table 2:** Incidence and types of CD19 CAR-T cell therapy-related fatalities from systematic review and meta-analysis.

**Variable**	**CD-19 Car-T (n=890)**	**Kymriah (n=201)**	**Yescarta (n=209)**	P*
Deaths, No. (%)	33 (3.71%)	7 (3.48%)	9 (4.31%)	0.667
Type of fatal toxic effect				
CRS	10 (30.30%)	0	2 (22.22%)	0.499
Nervous system disorders	6 (18.18%)	1 (14.29%)	1 (11.11%)	1
Infections and infestations	4 (12.12%)	1 (14.29%)	0	0.49
Blood and lymphatic system disorders	3 (9.09%)	2 (28.57%)	1 (11.11%)	0.617
Cardiac disorders	2 (6.06%)	1 (14.29%)	1 (11.11%)	1
Respiratory, thoracic and mediastinal disorders	2 (6.06%)	0	2 (22.22%)	0.499
Gastrointestinal disorders	2 (6.06%)	0	0	NA
Hepatobiliary disorders	1 (3.03%)	1 (14.29%)	0	1
unknown cause	3 (9.09%)	1 (14.29%)	2 (22.22%)	1

* Fatal toxic effects rates of Kymriah and Yescarta were compared using χ2 testing.

**Figure 1:**
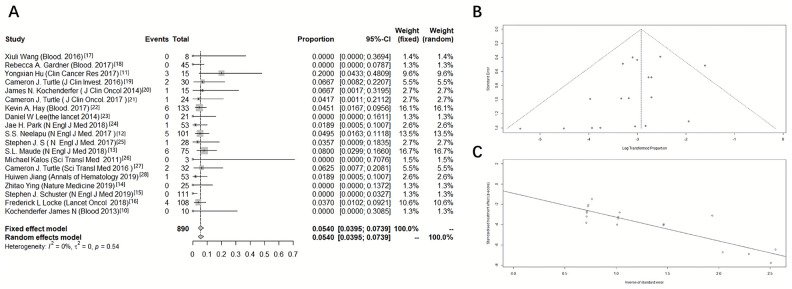
**Forest plot of the incidence of death due to fatal toxic effects associated with CD19 CAR-T cell therapy.** A meta-analysis was performed using R statistical software. Event rates and their corresponding 95% confidence intervals were estimated using both a fixed-effects model and a random-effects model. (**A**) Forest plot, (**B**) funnel plot, and (**C**) Egger test.

To describe the analytical results simply and intuitively, we created a forest plot, and the results showed that of the 890 participants, 33 had fatal toxic effects. Our meta-analysis showed that the incidence of fatal toxic effects related to death was 5.4%, and the 95% confidence interval of fatal toxic effects was between 3.95% and 7.39% (I^2^ = 0%, *P* = 0.54, Figure 1A).

To determine the presence of publication bias, a funnel plot and the Egger linear regression method were used. The funnel plot presented a general symmetry, and the P-value of the Egger test was 0.0622. The results of the above two methods indicated that there was no publication bias (Figure 1B, 1C).

**Figure 2:**
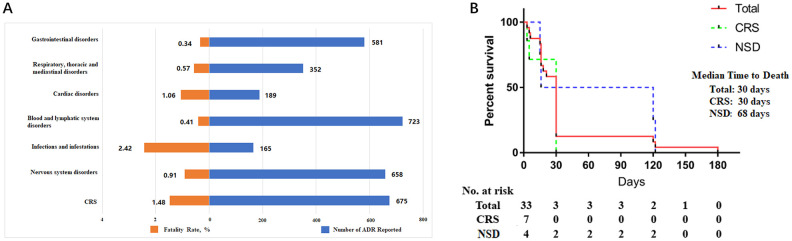
**Clinical characteristics of fatal toxic effects and time to death onset of fatal toxic effects.** (**A**) The number of cases (blue) and fatality rate (orange) for each class of toxic effect. (**B**) The time to death onset due to fatal toxic effects. Total (red), CRS: cytokine release syndrome (green), NSD: nervous system disorders (blue).

To determine the risk of fatality associated with particular toxic effects, we assessed fatality rates for different classes of toxic effects (Figure 2A). Infections and infestations appeared to present the highest risk of death, with 4 (2.41%) deaths among 165 cases. Toxic effects related to CRS, cardiac disorders, nervous system disorders, and respiratory disorders were associated with fatalities in 0.5% to 1.5% of the reported cases. Gastrointestinal and blood and lymphatic system disorders had the lowest reported fatality rates (0.34% and 0.41%, respectively). According to our meta-analysis, the toxic effect specific median time to death was 30, 30, and 68 days for total, CRS, and NSD (nervous system disorders), respectively (Figure 2B).

We noticed that the incidence of CRS and nervous system disorders was higher than that of other adverse reactions, so we carried a meta-analysis on the incidence of CRS and NSD over grade 3. For CRS, the incidence was more than 23% (I^2^ = 80%, *P* <0.01, Supplementary Figure 1A); for NSD, the incidence was more than 20% (I^2^ = 77%, *P* < 0.01, Supplementary Figure 1E). Since there was high heterogeneity, we performed a subgroup analysis, which indicated that age might be one of the reasons for the heterogeneity (Supplementary Figure 1B, 1F).

## DISCUSSION

Our work is the largest sample study on the toxicity of CD19 CAR-T treatment. Using the WHO database, we analyzed the toxic effects of Kymriah and Yescarta in real-world clinical applications. It showed that the proportion of immune system disorders, CRS, and NSD of Yescarta are higher than that of Kymriah. We systematically analyzed the data of published clinical trials. We found that the mortality caused by CD19 CAR-T-related lethal toxicity was 5.4%, which was higher than that of other immunotherapies (immune checkpoint therapy 0.36% - 1.08%) [10]. There is no significant difference between Kymriah and Yescarta in terms of fatal toxic effects.

We also found that although the incidence of toxic effects of the three groups was high, comparing all toxic effects, mortality was mostly related to infections and infestation, which is related to immunosuppression resulting from hematological malignancies. Although CRS and NSD are the most serious toxic effects, there are drugs to address CRS and NSD [6]; therefore, their associated mortality rate was low.

Both IL-6 and C-reactive protein (CRP) levels can predict CRS, and the level of IL-6 is positively correlated with the grade of CRS [11, 12]. We mainly use anti-IL-6 monoclonal antibodies to treat CRS, such as tocilizumab and siltuximab. Some studies have shown that therapeutic plasma exchange (TPE) may be the other option [13]. The use of glucocorticoids is also one of the main therapeutic methods, which are generally used to supplement anti-IL-6 therapy [14, 15].

However, studies have shown that glucocorticoid treatment of CRS may reduce the efficacy of CAR-T treatment, which is a controversial finding that needs further elucidation [16, 17].

NSD, also known as CAR-T cell-related encephalopathy syndrome (CRES), is related to the diffusion of cytokines into the nervous system [18]. Therefore, clinical studies have shown that anti-IL-6 drugs can also be used to treat CRES [6, 19]. However, there are also disputes regarding the choice of drugs. Some studies have shown that tocilizumab can alleviate neurotoxic effects while others have pointed out that it cannot cross the blood-brain barrier (BBB) [20, 21]. The mechanism of siltuximab is different and can effectively reduce the level of IL-6 and reduce neurotoxicity [22]. In addition, the Memorial Sloan Kettering Cancer Center MSKCC team found that anakinra, an anti-IL-1 drug, can be an option for reducing neurotoxicity [23]. To our knowledge, there is no official guideline on the treatment of nervous system toxicity. In clinical practice, it is necessary to have a high index of suspicion for fatal toxic effects during and after CD19 CAR-T cell therapy.

Contrary to our understanding of CAR-T therapy, our study found that infections and infestations are fatal toxic effects and should, therefore, receive more attention. When monitoring CRS and CRES, we should also be vigilant on the occurrence of infections and infestations, use antibiotics promptly, and reduce the infection risks of patients as much as possible during the treatment.

Also, with more patients receiving CAR-T treatment, we are increasingly learning some rare fatal toxic effects—for example, Lee DW et al. [19] found a case in which the CAR that should have been integrated into T cells was integrated into tumor cells, forming CAR-tumor cells, further increasing tumor drug resistance, and the patient died from the progression of the tumor. In addition to the report of CAR integration error causing an off-target effect or gene mutation, the JUNE team [24], the discoverer of CAR-T cell therapy, found CAR insertion into the TER2 gene of one patient, causing gene dysfunction, but this insertion brought a better therapeutic effect and indicated that CAR integration technology needs to be further improved. Currently, there are two types of transposition subsystems that have a good foreground, “Sleeping Beauty” transposition subsystems [25] and “piggy BAC” systems [26], which are expected to reduce the incidence of CAR integration errors.

Furthermore, as drugs are used to treat toxic effects, many researchers are also developing new ways to reduce the occurrence of toxic effects, such as integrating corresponding regulatory elements, inserting genes into CAR and so on, which can effectively prevent off-target and toxic effects while enhancing the therapeutic efficacy [27–31].

### Limitations

Our research has some limitations. Since the WHO database only recorded the number of toxic effects and did not include the number of deaths, we were unable to provide a real-world data analysis of the fatality rate. We hope that there will be a study with large sample size and more detailed real-world data to verify and analyze the fatal toxic effects associated with CAR-T cell therapy.

## CONCLUSIONS

Our study showed that the incidence of fatal toxic effects associated with CD19 CAR-T cell therapy is high. Infections and infestation, CRS, and nervous system disorders are the main causes of death. Whether it is clinical trials or WHO real-world data, the incidences of CRS and nervous system disorders were both higher than 50%. The overall mortality rate was 5.4% of all patients. However, the highest mortality rate, at 2.42% of patients, was related to infections and infestations. Most of the patients died within 30 days after treatment, indicating the need for clinicians to pay more attention to the monitoring and timely treatment of these fatal toxic effects, especially for infections and infestations.

## MATERIALS AND METHODS

### Vigilyze database

The study is based on adverse drug reactions reported in VigiBase (http://www.vigiaccess.org/), the WHO global Individual Safety Case Report (ISCR) database, originating from >130 countries. VigiBase is managed by the Uppsala Monitoring Center (UMC) and contains >16,000,000 ISCRs submitted by national pharmacovigilance centers since 1968. These reports originate from different sources, such as healthcare professionals, patients, and pharmaceutical companies, and are generally filed post-marketing [9].

We accessed Vigilyze on 30^th^ July 2019 and queried for Kymriah and Yescarta. We included all adverse drug reaction reports in which known CD19 CAR-T cell-related toxic effects occurred (Supplementary Table 1 in the Supplement). Patients with resolved toxic effects, unknown outcomes, or known/presumed cancer-related deaths were excluded. All the categories of toxic effects associated with CD19 CAR-T cell therapy are according to the rules of the Vigilyze Database.

### Meta-analysis

### Inclusion criteria

**Figure 3:**
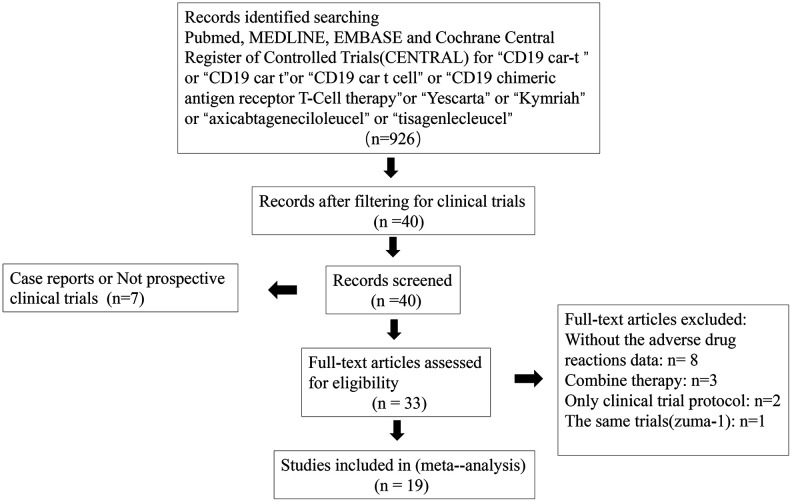
PRISMA diagram for articles selected for meta-analysis.

The study was registered in the PROSPERO (CRD42018112366). We identified records by searching PubMed, Medline, Embase and the Cochrane Central Register of Controlled Trials (CENTRAL) for “CD19 CAR-T” or “CD19 CAR T” or “CD19 CAR T cell” or “CD19 chimeric antigen receptor T-cell therapy” or “Yescarta” or “Kymriah” or “axicabtagene ciloleucel” or “tisagenlecleucel” on 30^th^ July 2019 (Figure 3). English-language clinical trials were included and spanned from 2013 to 2019.

### Exclusion criteria

All 926 studies were screened, and those that were clinical trials were included (n = 40), non-prospective clinical trials or case reports were all excluded (n = 7). Trials without adverse drug reaction data, those with combination therapy, those with only the clinical trial protocol provided, and those that were duplicates were excluded (n = 13). When two publications reported the same trial, the article with the longer follow-up time was selected (a phase II study that was initially reported and then subsequently reported with a prolonged follow-up, n=1).

The remaining trials (n = 19) were assessed individually for CD19 CAR-T cell therapy, Kymriah and Yescarta, the total number of patients treated, and the number and type of fatal toxic effects. The incidences of fatal toxic effects associated with CD19 CAR-T cell therapy were compared. Similarly, the types of toxic effects were compared (Supplementary Table 2, 3) [17, 19, 32–48]. All the categories of toxic effects associated with CD19 CAR-T cell therapy are according to the rules of the Vigilyze Database. The mortality data included in our cohort were from the patients who died from CD19 CAR-T cell therapy-related toxic effects.

### Statistics

Fatal toxic effect rates were compared using χ^2^ testing. Other clinical variables of interest were evaluated descriptively. Statistical analyses were performed in GraphPad Prism (version 7, GraphPad Software); the meta-analysis was performed using R statistical software (packages metafor and meta, R Foundation). Event rates and their corresponding 95% confidence intervals were estimated using both a fixed-effects model and a random-effects model. Forest plots were constructed to summarize the data for each analysis group with the incidence rate and to provide a visual analysis of studies evaluating fatal drug-related events.

## Supplementary Material

Supplementary Figure 1

Supplementary Table 1

Supplementary Table 2

Supplementary Table 3
