# Supplementary material for: A comprehensive analysis of the fatal toxic effects associated with CD19 CAR-T cell therapy
Source: Aging (Albany NY). 2020 Sep 24;12(18):18741–53. doi: 10.18632/aging.104058 (PMC7585129; doi:10.18632/aging.104058)
Supplement: Supplementary Figure 1 [file aging-12-104058-s001.pdf]

SUPPLEMENTARY FIGURE

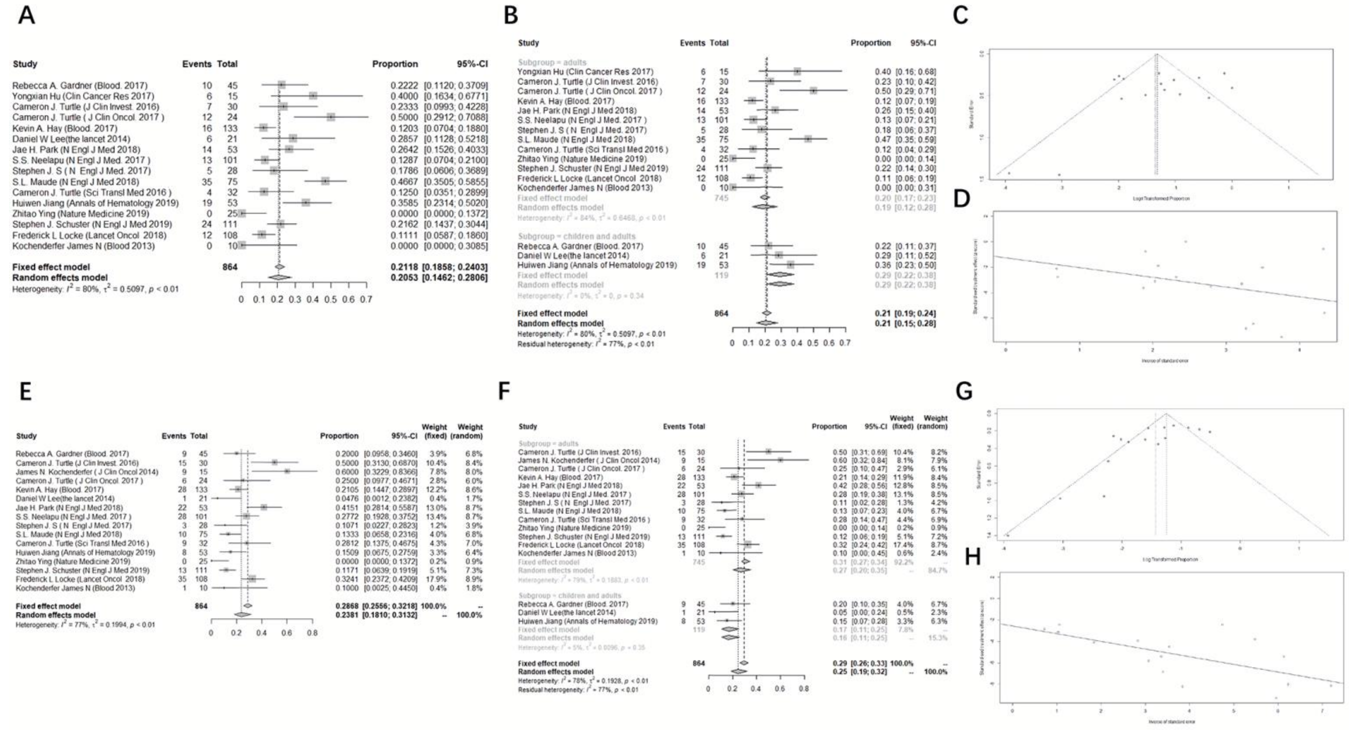

Supplementary Figure 1. Forest plot of the incidence of CRS and NSD associated with CD19 CAR-T cell therapy. (**A**) meta-analysis was performed using R statistical software. Event rates and their corresponding 95% confidence intervals were estimated using both a fixed-effects model and a random-effects model. (**A**–**D**) CRS: cytokine release syndrome. (**A**) Forest plot, (**B**) subgroup analysis, (**C**) funnel plot, (**D**) Egger test, (**E**–**H**) NSD: nervous system disorders, (**E**) forest plot, (**F**) subgroup analysis, (**G**) funnel plot, and (**H**) Egger test.
