# Supplementary material for: A comprehensive analysis of the fatal toxic effects associated with CD19 CAR-T cell therapy
Source: Aging (Albany NY). 2020 Sep 24;12(18):18741–53. doi: 10.18632/aging.104058 (PMC7585129; doi:10.18632/aging.104058)
Supplement: Supplementary Table 1 [file aging-12-104058-s002.docx]

**Supplementary Table 1. List of adverse drug reactions included in Vigilyze.**

| **Yescarta** | **Kymriah** |
| --- | --- |
| **Blood and lymphatic system disorders (137)** | **Blood and lymphatic system disorders (150)** |
| Neutropenia (55) | Febrile neutropenia (50) |
| Febrile neutropenia (34) | Neutropenia (30) |
| Thrombocytopenia (32) | Coagulopathy (24) |
| Pancytopenia (20) | Cytopenia (18) |
| Anaemia (16) | Disseminated intravascular coagulation (16) |
| Cytopenia (13) | Lymphopenia (15) |
| Bone marrow failure (5) | Pancytopenia (11) |
| Leukopenia (4) | Bone marrow failure (10) |
| Disseminated intravascular coagulation (3) | Anaemia (8) |
| Febrile bone marrow aplasia (3) | B-cell aplasia (8) |
| Coagulopathy (2) | Thrombocytopenia (7) |
| Lymphadenopathy (2) | Leukopenia (5) |
| Agranulocytosis (1) | Lymphadenopathy (5) |
| B-cell aplasia (1) | Splenomegaly (3) |
| Bone marrow disorder (1) | Febrile bone marrow aplasia (2) |
| Eosinophilia (1) | Hypofibrinogenaemia (2) |
| Lymphocytic infiltration (1) | Leukocytosis (2) |
| Lymphopenia (1) | Abdominal lymphadenopathy (1) |
| Platelet disorder (1) | Eosinophilia (1) |
| Spleen disorder (1) | Haemolytic anaemia (1) |
| **Cardiac disorders (119)** | Hyperleukocytosis (1) |
| Tachycardia (62) | Lymphocytosis (1) |
| Atrial fibrillation (27) | Normochromic normocytic anaemia (1) |
| Cardiac arrest (7) | Splenic infarction (1) |
| Cardiorenal syndrome (7) | Thrombocytosis (1) |
| Sinus tachycardia (6) | **Cardiac disorders (58)** |
| Pericardial effusion (5) | Tachycardia (33) |
| Bradycardia (4) | Left ventricular dysfunction (7) |
| Supraventricular tachycardia (3) | Atrial fibrillation (4) |
| Ventricular tachycardia (3) | Cardiac arrest (4) |
| Arrhythmia (2) | Arrhythmia (3) |
| Atrial flutter (2) | Cardiac failure (3) |
| Cardiopulmonary failure (2) | Pericardial effusion (3) |
| Cardio-respiratory arrest (2) | Sinus tachycardia (3) |
| Ventricular fibrillation (2) | Ventricular tachycardia (3) |
| Cardiac disorder (1) | Bradycardia (2) |
| Cardiac failure (1) | Cardiorenal syndrome (2) |
| Cardiac failure congestive (1) | Cardio-respiratory arrest (2) |
| Cardiomyopathy (1) | Pulseless electrical activity (2) |
| Cardiotoxicity (1) | Sinus bradycardia (2) |
| Myocardial infarction (1) | Atrial thrombosis (1) |
| Pulseless electrical activity (1) | Atrioventricular block first degree (1) |
| Stress cardiomyopathy (1) | Cardiac dysfunction (1) |
| Ventricular arrhythmia (1) | Cardiac failure congestive (1) |
| Ventricular failure (1) | Cardiomegaly (1) |
| Ventricular hypokinesia (1) | Mitral valve disease (1) |
| **Congenital, familial and genetic disorders (3)** | Myocarditis (1) |
| Aplasia (3) | Nodal rhythm (1) |
| **Endocrine disorders (7)** | Congenital, familial and genetic disorders (1) |
| Adrenal insufficiency (3) | Aplasia (1) |
| Inappropriate antidiuretic hormone secretion (2) | **Ear and labyrinth disorders (3)** |
| Diabetes insipidus (1) | Hypoacusis (2) |
| Hypercalcaemia of malignancy (1) | Tinnitus (1) |
| **Eye disorders (8)** | **Endocrine disorders (3)** |
| Diplopia (2) | Adrenal insufficiency (1) |
| Vision blurred (2) | Cushingoid (1) |
| Chorioretinal disorder (1) | Hypothyroidism (1) |
| Photophobia (1) | **Eye disorders (17)** |
| Retinal detachment (1) | Photophobia (3) |
| Vitreous floaters (1) | Visual impairment (3) |
| **Gastrointestinal disorders (72)** | Blindness (2) |
| Nausea (22) | Conjunctival haemorrhage (2) |
| Vomiting (18) | Mydriasis (2) |
| Dysphagia (13) | Papilloedema (2) |
| Diarrhoea (12) | Periorbital oedema (2) |
| Abdominal pain (8) | Pupil fixed (2) |
| Ascites (4) | Vision blurred (2) |
| Gastrointestinal haemorrhage (4) | Amaurosis fugax (1) |
| Intestinal perforation (3) | Blindness unilateral (1) |
| Colitis (2) | Diplopia (1) |
| Constipation (2) | Exophthalmos (1) |
| Abdominal discomfort (1) | Eye inflammation (1) |
| Anal incontinence (1) | Eye swelling (1) |
| Diverticular perforation (1) | Eyelid oedema (1) |
| Dyspepsia (1) | Eyelid ptosis (1) |
| Enteritis (1) | Iritis (1) |
| Frequent bowel movements (1) | Pupillary reflex impaired (1) |
| Gastrointestinal disorder (1) | Retinal artery occlusion (1) |
| Gastrointestinal oedema (1) | **Gastrointestinal disorders (64)** |
| Gastrointestinal pain (1) | Nausea (14) |
| Haematochezia (1) | Diarrhoea (13) |
| Haemorrhoidal haemorrhage (1) | Vomiting (8) |
| Hypoaesthesia oral (1) | Abdominal pain (5) |
| Ileus (1) | Pancreatitis (5) |
| Intestinal ischaemia (1) | Abdominal distension (4) |
| Lower gastrointestinal haemorrhage (1) | Gastrointestinal haemorrhage (4) |
| Noninfective sialoadenitis (1) | Abdominal compartment syndrome (3) |
| Oral dysaesthesia (1) | Ascites (3) |
| Peritoneal haemorrhage (1) | Dysphagia (3) |
| Pneumatosis intestinalis (1) | Oral pain (3) |
| Retroperitoneal haemorrhage (1) | Stomatitis (3) |
| Swollen tongue (1) | Abdominal discomfort (2) |
| **General disorders and administration site conditions (286)** | Colitis (2) |
| Pyrexia (214) | Gastrointestinal toxicity (2) |
| Fatigue (37) | Lip swelling (2) |
| Disease progression (27) | Mouth haemorrhage (2) |
| Malaise (22) | Neutropenic colitis (2) |
| Chills (17) | Proctalgia (2) |
| Death (15) | Abdominal mass (1) |
| Asthenia (10) | Abdominal pain lower (1) |
| Drug ineffective (9) | Abdominal tenderness (1) |
| Condition aggravated (5) | Abdominal wall mass (1) |
| Feeling abnormal (4) | Acute abdomen (1) |
| Pain (4) | Anal incontinence (1) |
| Gait disturbance (3) | Dry mouth (1) |
| Multiple organ dysfunction syndrome (3) | Enterocolitis (1) |
| Swelling (3) | Gastric dilatation (1) |
| Unevaluable event (3) | Gastrointestinal disorder (1) |
| Adverse event (2) | Gastrointestinal inflammation (1) |
| Disease recurrence (2) | Gastrointestinal oedema (1) |
| Feeling jittery (2) | Haematochezia (1) |
| Hyperthermia (2) | Ileus (1) |
| Inflammation (2) | Large intestinal obstruction (1) |
| Localised oedema (2) | Melaena (1) |
| No adverse event (2) | Mouth swelling (1) |
| Adverse drug reaction (1) | Odynophagia (1) |
| Chest pain (1) | Palatal disorder (1) |
| Feeling hot (1) | Palatal swelling (1) |
| Gait inability (1) | Pancreatic enlargement (1) |
| Generalised oedema (1) | Paraesthesia oral (1) |
| Granuloma (1) | Retroperitoneal mass (1) |
| Ill-defined disorder (1) | Swollen tongue (1) |
| Infusion site reaction (1) | Toothache (1) |
| Mucosal inflammation (1) | Upper gastrointestinal haemorrhage (1) |
| Oedema peripheral (1) | **General disorders and administration site conditions (259)** |
| Organ failure (1) | Pyrexia (171) |
| Therapy non-responder (1) | Drug ineffective (27) |
| Treatment failure (1) | Fatigue (23) |
| **Hepatobiliary disorders (11)** | Disease progression (15) |
| Hepatotoxicity (3) | Multiple organ dysfunction syndrome (12) |
| Hyperbilirubinaemia (3) | Death (11) |
| Hepatic failure (2) | Malaise (10) |
| Hepatocellular injury (2) | Pain (10) |
| Cholecystitis acute (1) | Therapy non-responder (9) |
| Cholestasis (1) | Chills (8) |
| Hepatic steatosis (1) | Asthenia (6) |
| Liver disorder (1) | Systemic inflammatory response syndrome (5) |
| **Immune system disorders (433)** | Disease recurrence (4) |
| **Cytokine release syndrome (426)** | Oedema peripheral (4) |
| Haemophagocytic lymphohistiocytosis (14) | Therapeutic response decreased (4) |
| Hypogammaglobulinaemia (6) | Treatment failure (4) |
| Allergic reaction to excipient (1) | Chest discomfort (3) |
| **Infections and infestations (99)** | Chest pain (3) |
| Pneumonia (11) | Feeling abnormal (3) |
| Infection (10) | Gait disturbance (2) |
| Sepsis (10) | Mucosal inflammation (2) |
| Clostridium difficile infection (7) | Nodule (2) |
| Septic shock (6) | Concomitant disease aggravated (1) |
| Systemic candida (6) | Discomfort (1) |
| Urinary tract infection (6) | Face oedema (1) |
| Staphylococcal infection (5) | Hernia (1) |
| Bacteraemia (4) | Hyperthermia (1) |
| Clostridium difficile colitis (4) | Ill-defined disorder (1) |
| Enterococcal infection (4) | Mass (1) |
| Candida infection (3) | Moaning (1) |
| Fungal infection (3) | Mucosal haemorrhage (1) |
| Pneumocystis jirovecii pneumonia (3) | Necrosis (1) |
| Sinusitis (3) | No adverse event (1) |
| Staphylococcal bacteraemia (3) | Non-cardiac chest pain (1) |
| Cytomegalovirus infection (2) | Organ failure (1) |
| Fungaemia (2) | Peripheral swelling (1) |
| Myelitis (2) | Pneumatosis (1) |
| Parainfluenzae virus infection (2) | Remission not achieved (1) |
| Pseudomonal bacteraemia (2) | Sense of oppression (1) |
| Rhinovirus infection (2) | Swelling (1) |
| Upper respiratory tract infection (2) | Unevaluable event (1) |
| Aspergillus infection (1) | **Hepatobiliary disorders (15)** |
| Bacterial infection (1) | Hepatic failure (4) |
| Candida sepsis (1) | Cholestasis (3) |
| Catheter site infection (1) | Hepatic function abnormal (2) |
| Cellulitis (1) | Hepatocellular injury (2) |
| Clostridium bacteraemia (1) | Hepatomegaly (2) |
| Device related sepsis (1) | Hyperbilirubinaemia (2) |
| Diverticulitis (1) | Liver disorder (2) |
| Encephalitis (1) | Gallbladder enlargement (1) |
| Escherichia pyelonephritis (1) | Hepatic cirrhosis (1) |
| Herpes simplex (1) | Hepatic necrosis (1) |
| Herpes zoster (1) | Hepatosplenomegaly (1) |
| Herpes zoster disseminated (1) | Hepatotoxicity (1) |
| Human ehrlichiosis (1) | Jaundice cholestatic (1) |
| Human herpesvirus 6 infection (1) | Liver injury (1) |
| Infection reactivation (1) | **Immune system disorders (261)** |
| Kidney infection (1) | **Cytokine release syndrome (237)** |
| Klebsiella sepsis (1) | Hypogammaglobulinaemia (38) |
| Lower respiratory tract infection fungal (1) | Haemophagocytic lymphohistiocytosis (11) |
| Lymph gland infection (1) | Cytokine storm (3) |
| Meningitis aseptic (1) | Anaphylactic reaction (2) |
| Meningoencephalitis herpetic (1) | Graft versus host disease in gastrointestinal tract (2) |
| Necrotising fasciitis (1) | Immune system disorder (2) |
| Neutropenic sepsis (1) | Acute graft versus host disease in intestine (1) |
| Oral candidiasis (1) | Acute graft versus host disease in liver (1) |
| Parvovirus infection (1) | Acute graft versus host disease in skin (1) |
| Pneumonia bacterial (1) | Drug hypersensitivity (1) |
| Pneumonia respiratory syncytial viral (1) | Graft versus host disease in liver (1) |
| Post procedural infection (1) | Graft versus host disease in skin (1) |
| Pseudomonas infection (1) | Kidney transplant rejection (1) |
| Rhinitis (1) | **Infections and infestations (112)** |
| Septic embolus (1) | Sepsis (12) |
| Sialoadenitis (1) | Staphylococcal infection (11) |
| Sinusitis fungal (1) | Infection (10) |
| Skin candida (1) | Candida infection (7) |
| Staphylococcal sepsis (1) | Pneumonia (7) |
| Stomatococcal infection (1) | Viral upper respiratory tract infection (7) |
| Streptococcal infection (1) | Clostridium difficile infection (6) |
| Systemic mycosis (1) | Device related infection (6) |
| Urinary tract infection pseudomonal (1) | Influenza (6) |
| Vascular device infection (1) | Bacteraemia (5) |
| Viraemia (1) | Clostridium difficile colitis (5) |
| Viral infection (1) | Fungal infection (5) |
| Viral upper respiratory tract infection (1) | Rhinovirus infection (5) |
| Wound infection (1) | Septic shock (5) |
| **Injury, poisoning and procedural complications (26)** | Viral sinusitis (5) |
| Toxicity to various agents (7) | Adenovirus infection (4) |
| Infusion related reaction (5) | Bronchopulmonary aspergillosis (4) |
| Accidental underdose (2) | Klebsiella infection (4) |
| Brain herniation (2) | Lung infection (4) |
| Fall (2) | Aspergillus infection (3) |
| Post procedural haematoma (2) | Encephalitis (3) |
| Product dose omission (2) | Mucormycosis (3) |
| Wrong technique in product usage process (2) | Sinusitis (3) |
| Corneal abrasion (1) | Staphylococcal bacteraemia (3) |
| Limb injury (1) | Upper respiratory tract infection (3) |
| Off label use (1) | Anal abscess (2) |
| Product dispensing error (1) | BK virus infection (2) |
| Skin injury (1) | Cellulitis (2) |
| Transfusion reaction (1) | Cytomegalovirus infection (2) |
| **Investigations (92)** | Enterobacter infection (2) |
| Transaminases increased (15) | Enterococcal infection (2) |
| White blood cell count decreased (13) | Escherichia infection (2) |
| C-reactive protein increased (12) | Gastroenteritis (2) |
| Platelet count decreased (12) | Nasopharyngitis (2) |
| Neutrophil count decreased (11) | Pseudomonal sepsis (2) |
| Serum ferritin increased (6) | Respiratory syncytial virus infection (2) |
| Aspartate aminotransferase increased (5) | Respiratory tract infection (2) |
| Ejection fraction decreased (5) | Stenotrophomonas infection (2) |
| Alanine aminotransferase increased (4) | Streptococcal infection (2) |
| Blood fibrinogen decreased (3) | Urinary tract infection (2) |
| Blood pressure decreased (3) | Acute sinusitis (1) |
| Liver function test increased (3) | Alpha haemolytic streptococcal infection (1) |
| Lymphocyte count decreased (3) | Catheter site infection (1) |
| Weight decreased (3) | Cellulitis of male external genital organ (1) |
| Blood count abnormal (2) | Cerebral toxoplasmosis (1) |
| Blood creatinine increased (2) | Conjunctivitis (1) |
| Blood lactate dehydrogenase increased (2) | Corynebacterium infection (1) |
| Body temperature increased (2) | Device related sepsis (1) |
| Culture urine positive (2) | Encephalitis viral (1) |
| Electrocardiogram QT prolonged (2) | Enterococcal bacteraemia (1) |
| International normalised ratio increased (2) | Enterovirus infection (1) |
| Oxygen saturation decreased (2) | Gastroenteritis salmonella (1) |
| Alanine aminotransferase abnormal (1) | Gastrointestinal bacterial infection (1) |
| Biopsy skin abnormal (1) | Gastrointestinal infection (1) |
| Blood bilirubin increased (1) | Herpes virus infection (1) |
| Blood calcium decreased (1) | Human herpesvirus 6 infection (1) |
| Blood culture (1) | Infection reactivation (1) |
| Blood culture negative (1) | Klebsiella sepsis (1) |
| Blood culture positive (1) | Localised infection (1) |
| Blood potassium decreased (1) | Lower respiratory tract infection fungal (1) |
| Blood pressure systolic decreased (1) | Lung infection pseudomonal (1) |
| Blood test abnormal (1) | Metapneumovirus infection (1) |
| Chest X-ray abnormal (1) | Necrotising fasciitis (1) |
| Coronavirus test positive (1) | Oral infection (1) |
| C-reactive protein abnormal (1) | Parainfluenzae virus infection (1) |
| Electroencephalogram abnormal (1) | Paronychia (1) |
| Haemoglobin decreased (1) | Pharyngitis streptococcal (1) |
| Hepatic enzyme increased (1) | Pneumocystis jirovecii pneumonia (1) |
| Human herpes virus 6 serology positive (1) | Pneumonia fungal (1) |
| Liver function test abnormal (1) | Pneumonia pseudomonal (1) |
| Neurological examination abnormal (1) | Pneumonia respiratory syncytial viral (1) |
| Ophthalmological examination abnormal (1) | Pseudomonas infection (1) |
| Pulse absent (1) | Pulmonary mycosis (1) |
| Pulse pressure increased (1) | Purulence (1) |
| Respiratory rate increased (1) | Rash pustular (1) |
| Serum ferritin abnormal (1) | Salmonellosis (1) |
| Sputum abnormal (1) | Skin infection (1) |
| Viral test positive (1) | Soft tissue infection (1) |
| Visual tracking test abnormal (1) | Staphylococcal sepsis (1) |
| **Metabolism and nutrition disorders (29)** | Stenotrophomonas sepsis (1) |
| Hyponatraemia (12) | Systemic mycosis (1) |
| Decreased appetite (3) | Tinea pedis (1) |
| Dehydration (2) | Toxoplasmosis (1) |
| Hypercalcaemia (2) | Urinary tract infection bacterial (1) |
| Hypernatraemia (2) | Urinary tract infection viral (1) |
| Hypoalbuminaemia (2) | Urosepsis (1) |
| Acidosis (1) | Viral infection (1) |
| Hypoglycaemia (1) | Viral sepsis (1) |
| Hypokalaemia (1) | **Injury, poisoning and procedural complications (21)** |
| Hypomagnesaemia (1) | Infusion related reaction (6) |
| Hypophagia (1) | Fall (3) |
| Hypovolaemia (1) | Accidental exposure to product (1) |
| Lactic acidosis (1) | Arthropod bite (1) |
| Malnutrition (1) | Contusion (1) |
| Metabolic acidosis (1) | Cranial nerve injury (1) |
| **Musculoskeletal and connective tissue disorders (25)** | Epiphyseal fracture (1) |
| Muscular weakness (8) | Optic nerve injury (1) |
| Back pain (5) | Procedural complication (1) |
| Myalgia (5) | Product administration error (1) |
| Arthralgia (2) | Product use in unapproved indication (1) |
| Bone pain (1) | Subdural haematoma (1) |
| Chest wall mass (1) | Thermal burn (1) |
| Musculoskeletal disorder (1) | Toxicity to various agents (1) |
| Neck pain (1) | Underdose (1) |
| Pain in extremity (1) | Vasoplegia syndrome (1) |
| Spinal stenosis (1) | **Investigations (173)** |
| Vertebral lesion (1) | Neutrophil count decreased (74) |
| Vertebral osteophyte (1) | Platelet count decreased (39) |
| **Neoplasms benign, malignant and unspecified (incl cysts and polyps) (49)** | White blood cell count decreased (28) |
| Diffuse large B-cell lymphoma (25) | C-reactive protein increased (15) |
| Diffuse large B-cell lymphoma recurrent (4) | Lymphocyte count decreased (12) |
| Lymphoma (3) | Serum ferritin increased (11) |
| Malignant neoplasm progression (3) | Heart rate increased (10) |
| Myelodysplastic syndrome (3) | Blood pressure decreased (9) |
| Non-Hodgkin's lymphoma (2) | Haemoglobin decreased (8) |
| Primary mediastinal large B-cell lymphoma (2) | Liver function test increased (7) |
| B-cell lymphoma (1) | Aspartate aminotransferase increased (6) |
| B-cell lymphoma recurrent (1) | Body temperature increased (6) |
| Double hit lymphoma (1) | Alanine aminotransferase increased (5) |
| High-grade B-cell lymphoma (1) | Blood bilirubin increased (5) |
| Lung neoplasm (1) | Blood fibrinogen decreased (5) |
| Lung neoplasm malignant (1) | International normalised ratio increased (5) |
| Malignant ascites (1) | Activated partial thromboplastin time prolonged (4) |
| Meningioma (1) | Blood creatinine increased (4) |
| Metastases to meninges (1) | Oxygen saturation decreased (4) |
| Neoplasm (1) | Staphylococcus test positive (4) |
| Neoplasm progression (1) | Blood glucose increased (3) |
| Plasmablastic lymphoma (1) | Blood lactate dehydrogenase increased (3) |
| **Nervous system disorders (422)** | Blood pressure increased (3) |
| Neurotoxicity (269) | Drug level decreased (3) |
| Encephalopathy (106) | Haematocrit decreased (3) |
| Aphasia (61) | Inflammatory marker increased (3) |
| Tremor (51) | Weight decreased (3) |
| Headache (41) | Blood albumin decreased (2) |
| Somnolence (36) | Blood alkaline phosphatase increased (2) |
| CAR T-cell-related encephalopathy syndrome (30) | Blood calcium decreased (2) |
| Dysgraphia (16) | Blood fibrinogen increased (2) |
| Memory impairment (15) | Blood immunoglobulin G decreased (2) |
| Seizure (13) | Blood lactic acid increased (2) |
| Depressed level of consciousness (9) | Blood potassium decreased (2) |
| Neurological symptom (9) | Blood pressure diastolic decreased (2) |
| Cerebrovascular accident (7) | Blood pressure systolic decreased (2) |
| Dizziness (7) | Blood pressure systolic increased (2) |
| Brain oedema (6) | B-lymphocyte count decreased (2) |
| Nervous system disorder (6) | Electrocardiogram QT prolonged (2) |
| Status epilepticus (5) | Electroencephalogram abnormal (2) |
| Cognitive disorder (4) | Fibrin D dimer increased (2) |
| 2019/8/6 VigiAccess | Red blood cell count decreased (2) |
| http://www.vigiaccess.org/ 8/12 | Respiratory rate increased (2) |
| Disturbance in attention (4) | Serum ferritin abnormal (2) |
| Dysarthria (4) | Transaminases increased (2) |
| Hemiparesis (4) | Alanine aminotransferase abnormal (1) |
| Syncope (4) | Alanine aminotransferase decreased (1) |
| Toxic encephalopathy (4) | Albumin globulin ratio decreased (1) |
| Amnesia (3) | Ammonia increased (1) |
| Facial paralysis (3) | Aspartate aminotransferase abnormal (1) |
| Hypoaesthesia (3) | Aspartate aminotransferase decreased (1) |
| Loss of consciousness (3) | Aspergillus test positive (1) |
| Central nervous system lesion (2) | Blood alkaline phosphatase decreased (1) |
| Cerebellar infarction (2) | Blood antidiuretic hormone abnormal (1) |
| Cerebellar syndrome (2) | Blood bilirubin decreased (1) |
| Coma (2) | Blood bilirubin unconjugated increased (1) |
| Dysmetria (2) | Blood count abnormal (1) |
| Essential tremor (2) | Blood creatinine decreased (1) |
| Motor dysfunction (2) | Blood culture positive (1) |
| Myoclonus (2) | Blood immunoglobulin E decreased (1) |
| Paraesthesia (2) | Blood magnesium decreased (1) |
| Presyncope (2) | Blood phosphorus decreased (1) |
| Slow speech (2) | Blood pressure abnormal (1) |
| Speech disorder (2) | Blood sodium decreased (1) |
| Subdural hygroma (2) | Blood thyroid stimulating hormone decreased (1) |
| Acalculia (1) | Blood triglycerides increased (1) |
| Akathisia (1) | Blood urea decreased (1) |
| Apraxia (1) | Blood urea increased (1) |
| Areflexia (1) | Blood urea nitrogen/creatinine ratio increased (1) |
| Ataxia (1) | Blood uric acid decreased (1) |
| Balance disorder (1) | Blood uric acid increased (1) |
| Basal ganglia stroke (1) | Carbon dioxide increased (1) |
| Brachial plexopathy (1) | CD4 lymphocytes decreased (1) |
| Brain stem infarction (1) | Cortisol decreased (1) |
| Cerebellar stroke (1) | C-reactive protein decreased (1) |
| Cerebral haematoma (1) | Cytogenetic analysis abnormal (1) |
| Cranial nerve paralysis (1) | Electrocardiogram QRS complex abnormal (1) |
| Dysaesthesia (1) | Electrocardiogram ST segment abnormal (1) |
| Dyscalculia (1) | Electrocardiogram T wave abnormal (1) |
| Horner's syndrome (1) | Gamma-glutamyltransferase increased (1) |
| Intention tremor (1) | Globulins increased (1) |
| Intracranial pressure increased (1) | Granulocyte count decreased (1) |
| Intraventricular haemorrhage (1) | Haemoglobin increased (1) |
| Language disorder (1) | Heart rate decreased (1) |
| Meningism (1) | Heart rate irregular (1) |
| Mental impairment (1) | Hepatic enzyme increased (1) |
| Metabolic encephalopathy (1) | Human herpes virus 6 serology positive (1) |
| Neurological decompensation (1) | Human metapneumovirus test positive (1) |
| Neuropathy peripheral (1) | Human rhinovirus test positive (1) |
| Noninfective encephalitis (1) | Immature granulocyte count increased (1) |
| Nystagmus (1) | Immunoglobulins decreased (1) |
| Paraplegia (1) | Intraocular pressure increased (1) |
| Partial seizures (1) | Lipase increased (1) |
| Poor quality sleep (1) | Liver function test abnormal (1) |
| Seizure like phenomena (1) | Lymphocyte count abnormal (1) |
| Sensory loss (1) | Mean cell haemoglobin increased (1) |
| Spinal cord oedema (1) | Mean cell volume increased (1) |
| Subarachnoid haemorrhage (1) | Monocyte count decreased (1) |
| Unresponsive to stimuli (1) | Neurological examination abnormal (1) |
| Vocal cord paresis (1) | Procalcitonin increased (1) |
| Product issues (3) | Protein total increased (1) |
| Device leakage (1) | Prothrombin time prolonged (1) |
| Product odour abnormal (1) | Red blood cell count increased (1) |
| Product packaging issue (1) | Red blood cell sedimentation rate decreased (1) |
| **Psychiatric disorders (109)** | Red cell distribution width increased (1) |
| Confusional state (55) | Respirovirus test positive (1) |
| Disorientation (26) | Roseolovirus test positive (1) |
| Mental status changes (25) | Rubulavirus test positive (1) |
| Agitation (18) | Serum ferritin decreased (1) |
| Delirium (9) | Thyroxine free increased (1) |
| Hallucination, visual (6) | T-lymphocyte count decreased (1) |
| Anxiety (5) | Troponin increased (1) |
| Hallucination (4) | Weight increased (1) |
| Restlessness (4) | White blood cell count (1) |
| Communication disorder (3) | White blood cell count abnormal (1) |
| Depression (3) | White blood cell count increased (1) |
| Aggression (2) | **Metabolism and nutrition disorders (49)** |
| Flat affect (2) | Decreased appetite (12) |
| Anhedonia (1) | Acidosis (9) |
| Bradyphrenia (1) | Fluid overload (9) |
| Bruxism (1) | Tumour lysis syndrome (8) |
| Depressed mood (1) | Hypokalaemia (6) |
| Enuresis (1) | Dehydration (5) |
| Hallucination, auditory (1) | Hyperphosphataemia (4) |
| Hallucinations, mixed (1) | Hypernatraemia (3) |
| Insomnia (1) | Hyperkalaemia (2) |
| Learning disorder (1) | Hypocalcaemia (2) |
| Mental disorder (1) | Hyponatraemia (2) |
| Mutism (1) | Lactic acidosis (2) |
| Nervousness (1) | Malnutrition (2) |
| Paranoia (1) | Metabolic acidosis (2) |
| Pressure of speech (1) | Cell death (1) |
| Stress (1) | Electrolyte imbalance (1) |
| Substance-induced psychotic disorder (1) | Feeding disorder (1) |
| Tachyphrenia (1) | Fluid retention (1) |
| Tangentiality (1) | Hypercalcaemia (1) |
| Thinking abnormal (1) | Hyperglycaemia (1) |
| **Renal and urinary disorders (39)** | Hyperuricaemia (1) |
| Incontinence (13) | Hypoalbuminaemia (1) |
| Acute kidney injury (9) | Hypomagnesaemia (1) |
| Renal failure (5) | Hypophosphataemia (1) |
| Urinary retention (3) | Iron overload (1) |
| Renal tubular necrosis (2) | **Musculoskeletal and connective tissue disorders (26)** |
| Dysuria (1) | Myalgia (7) |
| Hydronephrosis (1) | Arthralgia (4) |
| Micturition urgency (1) | Muscular weakness (4) |
| Nephropathy toxic (1) | Bone pain (3) |
| Oliguria (1) | Neck pain (3) |
| Pollakiuria (1) | Muscle rigidity (2) |
| Postrenal failure (1) | Musculoskeletal pain (2) |
| Renal disorder (1) | Musculoskeletal stiffness (2) |
| Renal impairment (1) | Pain in extremity (2) |
| Urinary bladder haemorrhage (1) | Back pain (1) |
| Urinary incontinence (1) | Bone lesion (1) |
| Reproductive system and breast disorders (1) | Haemarthrosis (1) |
| Epididymal disorder (1) | Muscle spasms (1) |
| **Respiratory, thoracic and mediastinal disorders (83)** | Muscle twitching (1) |
| Hypoxia (38) | Myopathy (1) |
| Pleural effusion (18) | Neck mass (1) |
| Tachypnoea (11) | Pain in jaw (1) |
| Dyspnoea (6) | Rhabdomyolysis (1) |
| Pulmonary oedema (5) | **Neoplasms benign, malignant and unspecified (incl cysts and polyps) (120)** |
| Pulmonary embolism (4) | Malignant neoplasm progression (60) |
| Respiratory failure (4) | Acute lymphocytic leukaemia recurrent (34) |
| Cough (3) | Diffuse large B-cell lymphoma (25) |
| Pneumonitis (3) | Acute lymphocytic leukaemia (18) |
| Acute respiratory distress syndrome (2) | B-cell type acute leukaemia (10) |
| Acute respiratory failure (2) | Acute myeloid leukaemia (5) |
| Atelectasis (2) | Diffuse large B-cell lymphoma recurrent (5) |
| Dysphonia (2) | Leukaemia recurrent (5) |
| Respiratory distress (2) | Second primary malignancy (4) |
| Apnoea (1) | Diffuse large B-cell lymphoma refractory (3) |
| Aspiration (1) | Lymphoma (3) |
| Emphysema (1) | B-cell lymphoma recurrent (2) |
| Lung disorder (1) | Chloroma (2) |
| Lung infiltration (1) | Pelvic neoplasm (2) |
| Oropharyngeal pain (1) | Transformation to acute myeloid leukaemia (2) |
| Pulmonary mass (1) | Acute lymphocytic leukaemia (in remission) (1) |
| Sputum discoloured (1) | Acute lymphocytic leukaemia refractory (1) |
| Stridor (1) | Acute myelomonocytic leukaemia (1) |
| **Skin and subcutaneous tissue disorders (15)** | Brain neoplasm (1) |
| Rash (5) | Follicle centre lymphoma, follicular grade I, II, III (1) |
| Angioedema (3) | Follicle centre lymphoma, follicular grade I, II, III recurrent (1) |
| Hyperhidrosis (2) | Histiocytic sarcoma (1) |
| Dermatitis psoriasiform (1) | Leukaemia (1) |
| Erythema (1) | Metastatic lymphoma (1) |
| Pruritus (1) | Myelodysplastic syndrome (1) |
| Rash pruritic (1) | Neoplasm swelling (1) |
| Skin lesion (1) | Non-Hodgkin's lymphoma (1) |
| Swelling face (1) | Prostate cancer (1) |
| Urticaria (1) | Thymoma (1) |
| **Social circumstances (5)** | **Nervous system disorders (153)** |
| Loss of personal independence in daily activities (3) | Neurotoxicity (64) |
| Refusal of treatment by patient (2) | Headache (37) |
| **Surgical and medical procedures (15)** | Encephalopathy (27) |
| Dialysis (6) | Somnolence (12) |
| Endotracheal intubation (3) | Seizure (11) |
| Haemodialysis (1) | Tremor (10) |
| Nephrostomy (1) | Depressed level of consciousness (8) |
| Renal replacement therapy (1) | Aphasia (6) |
| Stem cell transplant (1) | Facial paralysis (6) |
| Therapy cessation (1) | Status epilepticus (6) |
| **Vascular disorders (104)** | CAR T-cell-related encephalopathy syndrome (5) |
| Hypotension (85) | Cerebral haemorrhage (5) |
| Deep vein thrombosis (7) | Generalised tonic-clonic seizure (5) |
| Orthostatic hypotension (5) | Memory impairment (5) |
| Hypertension (3) | Neuropathy peripheral (5) |
| Haemodynamic instability (2) | Neurological symptom (4) |
| Shock (2) | Brain oedema (3) |
| Blood pressure fluctuation (1) | Clonus (3) |
| Flushing (1) | Cognitive disorder (3) |
| Haematoma (1) | Lethargy (3) |
| Hot flush (1) | Loss of consciousness (3) |
| Shock haemorrhagic (1) | Paraesthesia (3) |
| Superior vena cava syndrome (1) | Amnesia (2) |
| Thrombophlebitis (1) | Balance disorder (2) |
|  | Central nervous system haemorrhage (2) |
|  | Cerebellar syndrome (2) |
|  | Dizziness (2) |
|  | Dysarthria (2) |
|  | Dysgeusia (2) |
|  | Haemorrhage intracranial (2) |
|  | Motor dysfunction (2) |
|  | Nervous system disorder (2) |
|  | Slow response to stimuli (2) |
|  | Speech disorder (2) |
|  | Toxic encephalopathy (2) |
|  | Altered state of consciousness (1) |
|  | Central nervous system lesion (1) |
|  | Cerebellar infarction (1) |
|  | Cerebellar ischaemia (1) |
|  | Cerebral atrophy (1) |
|  | Cerebral infarction (1) |
|  | Cerebral ventricle dilatation (1) |
|  | Clumsiness (1) |
|  | Cranial nerve palsies multiple (1) |
|  | Disturbance in attention (1) |
|  | Dysaesthesia (1) |
|  | Dyskinesia (1) |
|  | Epileptic encephalopathy (1) |
|  | Extrapyramidal disorder (1) |
|  | Haemorrhagic stroke (1) |
|  | Hemiparesis (1) |
|  | Hyperaesthesia (1) |
|  | Hyperreflexia (1) |
|  | Hypersomnia (1) |
|  | Hyporesponsive to stimuli (1) |
|  | Idiopathic intracranial hypertension (1) |
|  | IIIrd nerve paralysis (1) |
|  | Incoherent (1) |
|  | Intracranial pressure increased (1) |
|  | Ischaemic cerebral infarction (1) |
|  | Leukoencephalopathy (1) |
|  | Metabolic encephalopathy (1) |
|  | Multiple sclerosis (1) |
|  | Myoclonic epilepsy (1) |
|  | Neuralgia (1) |
|  | Neuritis (1) |
|  | Neurological decompensation (1) |
|  | Non-24-hour sleep-wake disorder (1) |
|  | Noninfectious myelitis (1) |
|  | Optic neuritis (1) |
|  | Paralysis (1) |
|  | Paraparesis (1) |
|  | Phantom limb syndrome (1) |
|  | Posterior reversible encephalopathy syndrome (1) |
|  | Quadriplegia (1) |
|  | Sedation (1) |
|  | Sensory disturbance (1) |
|  | Slow speech (1) |
|  | Subarachnoid haemorrhage (1) |
|  | Unresponsive to stimuli (1) |
|  | Visual field defect (1) |
|  | **Product issues (14)** |
|  | Out of specification test results (13) |
|  | Thrombosis in device (1) |
|  | **Psychiatric disorders (52)** |
|  | Confusional state (25) |
|  | Delirium (10) |
|  | Mental status changes (10) |
|  | Disorientation (8) |
|  | Agitation (7) |
|  | Anxiety (2) |
|  | Irritability (2) |
|  | Mental disorder (2) |
|  | Boredom (1) |
|  | Fear (1) |
|  | Hallucination (1) |
|  | Hallucination, visual (1) |
|  | Intensive care unit delirium (1) |
|  | Mania (1) |
|  | Negativism (1) |
|  | Personality change (1) |
|  | Rapid eye movement sleep behaviour disorder (1) |
|  | Sleep disorder (1) |
|  | Stress (1) |
|  | Tic (1) |
|  | Renal and urinary disorders (39) |
|  | Acute kidney injury (24) |
|  | Renal failure (6) |
|  | Renal impairment (4) |
|  | Haematuria (2) |
|  | Incontinence (2) |
|  | Renal tubular necrosis (2) |
|  | Anuria (1) |
|  | Chromaturia (1) |
|  | Hydronephrosis (1) |
|  | Kidney enlargement (1) |
|  | Oliguria (1) |
|  | Polyuria (1) |
|  | Urinary hesitation (1) |
|  | Urinary incontinence (1) |
|  | Urinary tract obstruction (1) |
|  | Reproductive system and breast disorders (2) |
|  | Testicular necrosis (1) |
|  | Testicular swelling (1) |
|  | **Respiratory, thoracic and mediastinal disorders (89)** |
|  | Hypoxia (41) |
|  | Dyspnoea (11) |
|  | Respiratory failure (11) |
|  | Cough (7) |
|  | Pulmonary oedema (7) |
|  | Respiratory distress (7) |
|  | Tachypnoea (6) |
|  | Acute respiratory failure (5) |
|  | Pleural effusion (5) |
|  | Acute respiratory distress syndrome (3) |
|  | Epistaxis (3) |
|  | Pulmonary embolism (3) |
|  | Rhinorrhoea (3) |
|  | Apnoea (2) |
|  | Atelectasis (2) |
|  | Oropharyngeal pain (2) |
|  | Pharyngeal haemorrhage (2) |
|  | Pneumonitis (2) |
|  | Pulmonary haemorrhage (2) |
|  | Respiratory alkalosis (2) |
|  | Respiratory disorder (2) |
|  | Aspiration (1) |
|  | Asthma (1) |
|  | Bronchial oedema (1) |
|  | Dysphonia (1) |
|  | Haemoptysis (1) |
|  | Hypocapnia (1) |
|  | Lung consolidation (1) |
|  | Nasal flaring (1) |
|  | Pneumonia aspiration (1) |
|  | Pulmonary mass (1) |
|  | Pulmonary pneumatocele (1) |
|  | Pulmonary toxicity (1) |
|  | Respiratory arrest (1) |
|  | Wheezing (1) |
|  | **Skin and subcutaneous tissue disorders (23)** |
|  | Geographical distribution |
|  | Rash (7) |
|  | Erythema (3) |
|  | Rash pruritic (2) |
|  | Alopecia (1) |
|  | Blood blister (1) |
|  | Dermatitis exfoliative generalised (1) |
|  | Ecchymosis (1) |
|  | Hyperhidrosis (1) |
|  | Night sweats (1) |
|  | Nodular rash (1) |
|  | Petechiae (1) |
|  | Pruritus (1) |
|  | Purpura (1) |
|  | Rash erythematous (1) |
|  | Rash generalised (1) |
|  | Skin discolouration (1) |
|  | Skin hyperpigmentation (1) |
|  | Skin hypertrophy (1) |
|  | Skin lesion (1) |
|  | Skin mass (1) |
|  | Skin reaction (1) |
|  | Skin sensitisation (1) |
|  | Skin warm (1) |
|  | Urticaria (1) |
|  | Social circumstances (1) |
|  | Loss of personal independence in daily activities (1) |
|  | **Vascular disorders (99)** |
|  | Hypotension (79) |
|  | Hypertension (9) |
|  | Haemodynamic instability (5) |
|  | Pallor (4) |
|  | Capillary leak syndrome (3) |
|  | Hypoperfusion (3) |
|  | Haemorrhage (2) |
|  | Peripheral coldness (2) |
|  | Thrombosis (2) |
|  | Capillary disorder (1) |
|  | Deep vein thrombosis (1) |
|  | Flushing (1) |
|  | Infarction (1) |
|  | Vascular compression (1) |
|  | Venoocclusive disease (1) |
|  | Visceral congestion (1) |
