# Supplementary material for: A comprehensive analysis of the fatal toxic effects associated with CD19 CAR-T cell therapy
Source: Aging (Albany NY). 2020 Sep 24;12(18):18741–53. doi: 10.18632/aging.104058 (PMC7585129; doi:10.18632/aging.104058)
Supplement: Supplementary Table 2 [file aging-12-104058-s003.docx]

**Supplementary Table 2. List of adverse drug reactions included in clinic trials.**

| **Toxicity** |
| --- |
| **CRS (675)** |
| **General disorders and administration site conditions（758）** |
| Pyrexia (283) |
| Fatigue (160) |
| Chills (95) |
| disease progression (54) |
| Oedema peripheral (38) |
| Disease progression (27) |
| Pain (21) |
| Fever (16) |
| Asthenia (9) |
| Malaise (7) |
| Edema limbs (7) |
| Gait disturbance (5) |
| Tumor lysis syndrom (5) |
| Multiple organ dysfunction syndrome (3) |
| Generalised oedema (3) |
| Tumour pain (3) |
| Localised oedema (3) |
| Non-cardiac chest pain (2) |
| Chest pain (1) |
| Dyskinesia (1) |
| Ear discomfort (1) |
| Ear pain (1) |
| Excessive cerumen production (1) |
| Face oedema (1) |
| Groin pain (1) |
| Hypoacusis (1) |
| Hypothermia (1) |
| Limb discomfort (1) |
| Mood altered (1) |
| Night sweats (1) |
| Oedema (1) |
| Scrotal oedema (1) |
| Toothache (1) |
| Peripheral swelling (1) |
| Head titubation (1) |
| **Nervous system disorders (658)** |
| Neurotoxicity (274) |
| Headache (66) |
| Encephalopathy (56) |
| Tremor（43） |
| confusional state (39) |
| Dizziness (25) |
| Aphasia (25) |
| Somnolence (20) |
| Memory impairment (10) |
| Dysarthria (6) |
| Seizure (6) |
| disturbance in attention (6) |
| Ataxia (5) |
| Speech disorder (5) |
| dysphagia (5) |
| Lethargy (4) |
| delirium (3) |
| mental status changes (3) |
| Presyncope (3) |
| Amnesia (3) |
| Myoclonus (3) |
| Cognitive disturbance (3) |
| Depressed level of consciousness (2) |
| Dyscalculia (2) |
| Hemiparesis (2) |
| Neuropathy peripheral (2) |
| agitation (2) |
| dyskinesia (2) |
| Facial paralysis (2) |
| Intraventricular haemorrhage (2) |
| Loss of consciousness (2) |
| Paraesthesia (2) |
| Stupor (2) |
| Syncope (2) |
| Nervous system disorders-Other (2) |
| pontine hemorrhage (1) |
| Hydrocephalus (1) |
| Movements involuntary (1) |
| Peripheral motor neuropathy (1) |
| Spasticity (1) |
| irritability (1) |
| metabolic encephalopathy (1) |
| abnormal thinking (1) |
| Procedural headache (1) |
| Cerebellar infarction (1) |
| Coordination abnormal (1) |
| Facial paresis (1) |
| Hypersomnia (1) |
| Hypoaesthesia (1) |
| Leukoencephalopathy (1) |
| Meningism (1) |
| Papilloedema (1) |
| Peripheral sensory neuropathy (1) |
| Vagus nerve disorder (1) |
| **Investigations (1175)** |
| White-cell count decreased (157) |
| Neutrophil count decreased (149) |
| Decreased platelet count（109） |
| Hypocalcaemia (83) |
| Hypokalemia (78) |
| Hyponatraemia (71) |
| Hypophosphataemia（69） |
| Aspartate aminotransferase increased (50) |
| Decreased lymphocyte count (66) |
| Alanine aminotransferase increased (45) |
| Hypoalbuminemia (41) |
| Hypomagnesaemia (39) |
| Hypokalaemia (36) |
| Blood bilirubin increased (33) |
| Blood creatinine increased (26) |
| Hyperglycaemia (20) |
| Weight decreased (17) |
| APTT prolonged（15） |
| Fibrinogen decreased（15） |
| Hyperkalaemia (7) |
| Blood alkaline phosphatase increased (6) |
| CPK increased (4) |
| Ejection fraction decreased (4) |
| Hypermagnesaemia (3) |
| Blood immunoglobulin G Decreased (2) |
| Hypercalcaemia (2) |
| Hyperuricaemia (2) |
| Serum ferritin increased (2) |
| CD4 lymphocyte decreased (2) |
| Cholesterol high (2) |
| Troponin increased (2) |
| hemophagocytic lymphohistiocytosis（1） |
| Blood albumin decreased (1) |
| Blood magnesium decreased (1) |
| Blood urea increased (1) |
| Blood uric acid increased (1) |
| C-reactive protein increased (1) |
| Hepatic enzyme increased (1) |
| Hyperalbuminaemia (1) |
| Liver function test abnormal (1) |
| Liver function test increased (1) |
| Oxygen saturation decreased (1) |
| Prothrombin time prolonged (1) |
| Respiratory rate increased (1) |
| Transaminases increased (1) |
| Troponin I increased (1) |
| Troponin T increased (1) |
| Lymphocyte count increased (1) |
| Weight gain (1) |
| **Blood and lymphatic system disorders (723)** |
| Anaemia (248) |
| Febrile neutropenia (120) |
| Neutropenia (156) |
| Thrombocytopenia (111) |
| Coagulopathy (31) |
| Disseminated intravascular coagulation (18) |
| Leukopenia (20) |
| Lymphopenia (10) |
| Pancytopenia (3) |
| Bone marrow failure (1) |
| Hernia (1) |
| Thrombocytosis (1) |
| Blood and lymphatic system disorders-Other (1) |
| Prolonged activated partial thromboplastin time（1） |
| Leukocytosis (1) |
| **Cardiac disorders (189)** |
| Tachycardia (96) |
| Sinus tachycardia (25) |
| Atrial fibrillation (9) |
| Sinus bradycardia (7) |
| Atrial flutter (6) |
| Ventricular arrhythmia (7) |
| Cardiac arrest (10) |
| Ventricular tachycardia (3) |
| Acute left ventricular failure (2) |
| Arrhythmia (2) |
| Supraventricular tachycardia (2) |
| Atrioventricular block (1) |
| Bradycardia (1) |
| Bundle branch block right (1) |
| Cardiomegaly (1) |
| Electrocardiogram QT prolonged (1) |
| Extrasystoles (1) |
| Heart rate irregular (1) |
| Orthopnoea (1) |
| Palpitations (1) |
| Supraventricular extrasystoles (1) |
| Atrial fibrillation (1) |
| Cardiac failure (2) |
| Chest pain -cardiac (1) |
| Restrictive cardiomyopathy (1) |
| QTc prolongation（1） |
| Left ventricular systolic Dysfunction（1） |
| cardiac arrhythmia（1) |
| Cardiac ventricular dysfunction (1) |
| Left ventricular dysfunction（1） |
| **Vascular disorders (246)** |
| Hypotension (192) |
| Hypertension (26) |
| Thrombosis (5) |
| Deep vein thrombosis (3) |
| Orthostatic hypotension (3) |
| Flushing (2) |
| Catheter site haematoma (1) |
| Catheter site pain (1) |
| Catheter site swelling (1) |
| Diastolic hypotension (1) |
| Embolism venous (1) |
| Haematoma (1) |
| Shock (1) |
| Splenic infarction (1) |
| Splenic vein thrombosis (1) |
| Thrombosis in device (1) |
| Capillary leak syndrome (1) |
| Hot flashes (1) |
| vascular leak syndrome（1） |
| Thromboembolic event (1) |
| Vascular disorders Other (1) |
| **Respiratory, thoracic and mediastinal disorders（352）** |
| Cough（63） |
| Hypoxia（73） |
| Respiratory failure（21） |
| Dyspnea（47） |
| Pleural effusion (36) |
| Pulmonary oedema (27) |
| Upper respiratory tract infection (13) |
| Oropharyngeal pain (8) |
| Nasal congestion (4) |
| Atelectasis (4) |
| Pneumonitis（4） |
| Productive cough (3) |
| Tachypnoea (3) |
| Respiratory thoracic and mediastinal disorders-Other (3) |
| Acute respiratory distress syndrome (2) |
| Pulmonary embolism (2) |
| Respiratory syncytial virus infection (2) |
| Aspiration (2) |
| Dysphonia (2) |
| Rhinorrhoea (2) |
| Sinus congestion (2) |
| Acute respiratory failure (1) |
| Aspiration (1) |
| Breath sounds abnormal (1) |
| Bronchitis (1) |
| Bronchopulmonary aspergillosis (1) |
| Haemoptysis (1) |
| Laryngeal haemorrhage (1) |
| Nasal dryness (1) |
| Nasopharyngitis (1) |
| Nasogastric output abnormal (1) |
| Obstructive airways disorder (1) |
| Pneumothorax (1) |
| Pulmonary congestion (1) |
| Pulmonary haemorrhage (1) |
| Pulmonary hypertension (1) |
| Rales (1) |
| Reexpansion pulmonary oedema (1) |
| Respiratory distress (1) |
| Sinus pain (1) |
| Upper-airway cough syndrome (1) |
| Wheezing (1) |
| Chest discomfort (1) |
| Tonsillar hypertrophy (1) |
| Allergic rhinitis (1) |
| Bronchial stricture (1) |
| Hoarseness (1) |
| Laryngeal edema (1) |
| Retinoic acid syndrome (1) |
| Sleep apnea (1) |
| **Psychiatric disorders (34)** |
| Confusion (7) |
| Anxiety (6) |
| Hallucination, visual (5) |
| Delirium (4) |
| Hallucinations (4) |
| Insomnia (4) |
| Mental status changes (4) |
| **Gastrointestinal disorders (581)** |
| Nausea（163） |
| Diarrhoea （147） |
| Vomiting（89） |
| Constipation (90) |
| Abdominal pain (21) |
| Abdominal distension (7) |
| Hiccups (7) |
| Dysphagia (6) |
| Dyspepsia (4) |
| Abdominal discomfort (3) |
| Ascites (3) |
| Rectal haemorrhage (3) |
| Abdominal distension (3) |
| Gastrointestinal disorders-Other (3) |
| Anal incontinence (2) |
| Gastritis (2) |
| Pancreatitis (2) |
| Mucositis oral (2) |
| Mucosal inflammation (2) |
| Abdominal X-ray (1) |
| Abdominal hernia (1) |
| Abdominal pain lower (1) |
| Colitis (1) |
| Cytomegalovirus enteritis (1) |
| Enteritis (1) |
| Ileus (1) |
| Neurogenic bowel (1) |
| Oesophageal fistula (1) |
| Oesophageal pain (1) |
| Oral discomfort (1) |
| Proctalgia (1) |
| Tongue disorder (1) |
| Flatulence (1) |
| Gastrooesophageal reflux disease (1) |
| Haemorrhoids (1) |
| Dysgeusia (1) |
| a fatal gastrointestinal (GI) hemorrhage (1) |
| alimentary tract hemorrhage (1) |
| Appetite decrease (1) |
| Intra-abdominal hemorrhage (1) |
| Small intestinal obstruction (1) |
| **Eye disorders (28)** |
| Vision blurred (7) |
| Dry eye (2) |
| Eye disorders-Other, specify (2) |
| Floaters (2) |
| Conjunctivitis (2) |
| Photophobia (2) |
| Blepharospasm (1) |
| Eye disorder (1) |
| Eye pain (1) |
| Keratitis (1) |
| Periorbital oedema (1) |
| Retinal tear (1) |
| Scleral haemorrhage (1) |
| Vitreous floaters (1) |
| defective visual field (1) |
| Papilledema (1) |
| Optic nerve disorder (1) |
| **Renal and urinary disorders (95)** |
| Acute kidney injury (33) |
| Creatinine increased（17） |
| Urinary incontinence (8) |
| Sinusitis (7) |
| Dysuria (6) |
| Urinary retention (4) |
| Pollakiuria (3) |
| Hematuria (2) |
| Renal and urinary disorders- Other (2) |
| Micturition urgency (2) |
| Oliguria (2) |
| Nocturia (1) |
| Polyuria (1) |
| Renal impairment (1) |
| Urinary tract infection bacterial (1) |
| Urinary tract obstruction (1) |
| Urine output decreased (1) |
| Renal failure（1） |
| Urinary urgency (1) |
| Urinary frequency (1) |
| **Metabolism and nutrition disorders (390)** |
| Decreased appetite (148) |
| Hyperglycaemia (53) |
| Hypernatraemia (18) |
| Weight decreased (17) |
| Dehydration (14) |
| hypertriglyceridemia (12) |
| Hypophosphatemia (15) |
| Hypocalcemia (15) |
| Hypokalemia (24) |
| Hypoalbuminemia (9) |
| Hypomagnesemia (9) |
| hyperuricemia (9) |
| Dry mouth (13) |
| Metabolic acidosis (5) |
| Malnutrition (4) |
| Hypoglycaemia (3) |
| Acidosis (3) |
| Anorexia (3) |
| Hypercalcemia (3) |
| Hypermagnesemia (2) |
| Lactic acidosis (2) |
| Weight increased (1) |
| Acidosis (1) |
| Hypertriglyceridaemia (1) |
| Hypervolaemia (1) |
| Hypouricaemia (1) |
| Metabolic alkalosis (1) |
| Tumour lysis syndrome (1) |
| Fluid overload (1) |
| Hypoalbuminemia (1) |
| **Ear and labyrinth disorders (3)** |
| Hearing impaired (1) |
| Vertigo (2) |
| **Immune system disorders Total (22)** |
| Hypogammaglobulinaemia (16) |
| Rhinitis allergic (3) |
| Allergic reaction (2) |
| Haemophagocytic lymphohistiocytosis (1) |
| **Infections and infestations (165)** |
| Upper respiratory tract infection (19) |
| Lung infection (14) |
| Urinary tract infection (17) |
| Infections and infestations. Other (11) |
| Pneumonia (11) |
| Bacteraemia (8) |
| Herpes zoster (8) |
| Clostridium difficile infection (5) |
| Clostridium difficile colitis (4) |
| Sinusitis (4) |
| Encephalitis (3) |
| Influenza (3) |
| Rhinovirus infection (2) |
| Septic shock (2) |
| Staphylococcal bacteremia (2) |
| Flu like symptoms (2) |
| Bronchial infection (2) |
| Gum infection (2) |
| Candida infection (2) |
| Escherichia bacteraemia (2) |
| Herpes simplex (2) |
| Oral candidiasis (2) |
| Post herpetic neuralgia (2) |
| Rhinitis (2) |
| Bacterial sepsis (1) |
| Cellulitis (1) |
| Cytomegalovirus infection (1) |
| Cytomegalovirus viraemia (1) |
| Device related infection (1) |
| Device related sepsis (1) |
| Fungal skin infection (1) |
| Herpes zoster oticus (1) |
| Human herpesvirus 6 infection (1) |
| Infusion site infection (1) |
| Klebsiella infection (1) |
| Localised infection (1) |
| Osteomyelitis (1) |
| Parainfluenzae virus infection (1) |
| Parvovirus infection (1) |
| Pneumonia klebsiella (1) |
| Pneumonia staphylococcal (1) |
| Respiratory tract infection viral (1) |
| Rhinovirus infection (1) |
| Salmonellosis (1) |
| Sepsis (1) |
| Tongue fungal infection (1) |
| Viral upper respiratory tract infection (1) |
| Vulvovaginal candidiasis (1) |
| Wound infection (1) |
| Abdominal infection (1) |
| Mucosal infection (1) |
| Papulopustular rash (1) |
| Rash pustular (1) |
| Sepsis (1) |
| Skin infection (1) |
| Small intestine infection (1) |
| Vaginal infection (1) |
| Systemic mycosis (1) |
| **Skin and subcutaneous tissue disorders (81)** |
| Social circumstances- Other, specify (GVHD) (15) |
| Skin and subcutaneous tissue disorders-Other (13) |
| Rash (5) |
| Rash maculo-papular (4) |
| Oral herpes (3) |
| Skin abrasion (3) |
| Swelling (3) |
| Alopecia (4) |
| Purpura (2) |
| Skin ulceration (2) |
| Dry skin (2) |
| Hyperhidrosis (2) |
| Pain of skin (2) |
| Pruritus generalised (2) |
| Chapped lips (1) |
| Contusion (1) |
| Ecchymosis (1) |
| Erythema (1) |
| Graft versus host disease in skin (1) |
| Increased tendency to bruise (1) |
| Lip dry (1) |
| Lip swelling (1) |
| Livedo reticularis (1) |
| Mouth ulceration (1) |
| Papule (1) |
| Rash erythematous (1) |
| Skin lesion (1) |
| Skin ulcer (1) |
| Tinea versicolour (1) |
| Erythema multiforme (1) |
| Erythroderma (1) |
| Skin itch (1) |
| Pruritus (1) |
| **Musculoskeletal and connective tissue disorders (126)** |
| Back pain (28) |
| Myalgia (16) |
| Pain in extremity (15) |
| Muscular weakness (17) |
| Arthralgia (16) |
| Neck pain (10) |
| Musculoskeletal pain (5) |
| Musculoskeletal and connective tissue disorder- Other (4) |
| Bone pain (3) |
| Muscle spasms (2) |
| Muscle spasticity (1) |
| Musculoskeletal chest pain (1) |
| Pelvic pain (1) |
| Sternal fracture (1) |
| Torticollis (1) |
| Non-cardiac chest pain (1) |
| Flank pain (1) |
| Flank pain (1) |
| Joint rang e of motion decreased (1) |
| Osteoporosis (1) |
| **Reproductive system and breast disorders (7)** |
| Perineal pain (1) |
| Vaginal haemorrhage (2) |
| Amenorrhoea (1) |
| Vaginal discharge (1) |
| Dyskinesia (1) |
| Erectile dysfunction (1) |
| **Hepatobiliary disorders (3)** |
| Hepatitis B reactivation (1) |
| Hyperbilirubinaemia (1) |
| Hepatotoxicity **(1)** |
| **Neoplasms benign, malignant and unspecified (incl cysts and polyps) (12)** |
| B-cell lymphoma (5) |
| Myelodysplastic syndrome (2) |
| Basal cell carcinoma (1) |
| Carcinoma in situ (1) |
| Neck mass (1) |
| Retention cyst (1) |
| Squamous cell carcinoma (1) |
| **Endocrine disorders (2)** |
| Inappropriate antidiuretic hormone secretion (1) |
| Amenorrhoea (1) |
| **unknown causes (5)** |
