# Supplementary material for: A comprehensive analysis of the fatal toxic effects associated with CD19 CAR-T cell therapy
Source: Aging (Albany NY). 2020 Sep 24;12(18):18741–53. doi: 10.18632/aging.104058 (PMC7585129; doi:10.18632/aging.104058)
Supplement: Supplementary Table 3 [file aging-12-104058-s004.docx]

| **Supplementary Table 3. Clinical trials and the characteristics of patients** | | | | | | | | | | | | | |
| --- | --- | --- | --- | --- | --- | --- | --- | --- | --- | --- | --- | --- | --- |
| study | study type | Ages | tumor types | Treatment | generation of Car | Infusion times | Phase | # of total patients | # deaths | Death types | Grade >3 CRS and NSD | Time to Onset of Death |  |
| Kochenderfer James N, Blood. 2013;122(25):4129-4139 | Non-Randomized+Sequential Assignment | 44-66 | B-cell malignancies persisting after allogeneic hematopoietic stem cell transplantation | donor derived allogeneic anti-CD19-CAR T cells | NA | NA | phase1 | 10 | 0 | NA | CRS:grade 3: 0%(0/10); NSD:grade 3: 10%(1/10) | NA |  |
| Yongxian Hu, Clinical Cancer Research, 2017, 23(13): 3297-3306. | Single arm | ＜60 | Relapsed/Refractory Acute Lymphocytic Leukemia | CD19 CAR–T cells | NA | NA | Phase I | 15 | 3 | severe infection | CRS:grade 3 40%(6/15); NSD: NA | 6d;16d;180d |  |
| S.S. Neelapu,N Engl J Med. 2017;377(26)_2531-2544. | Single Group Assignment | adults | Refractory large B-cell lymphoma | axicabtagene ciloleucel (axi-cel), an autologous anti-CD19 chime- Theric antigen receptor (CAR) T-cell | NA | NA | phase 2 | 101 | 5 | General disorders and administration site the cytokine release syndrome (2)  Respiratory, thoracic and mediastinal disorders (1) Two unknown causes (2) | CRS: grade >3: 13% (13 / 101); NSD:grade >3: 28%( 28 / 101) | NA |  |
| S.L. Maude N Engl J Med 2018;378:439-48 | single-cohort | adults | CD19+ relapsed or refractory B-cell ALL | tisagenlecleucel | NA | 1 | phase 2 | 75 | 6 | cerebral hemorrhage (1)15d lymphopenia and neutropenia (1) unknown causes (1); lymphopenia and Neutropenia (1); Hepatotoxicity (1); pneumonia:(1); | CRS: grade >3 47% (35 / 75) NSD: grade >3: 13% (10 / 75) | 15d, 30d; within 30d; after 30d; |  |
| Ying Z, Nature Medicine. 2019: 1. | Single Group Assignment | adults | refractory B cell lymphoma | anti-CD19 CAR-T | The second-generation | NA | phase 1 | 25 | 0 | NA | NA | NA |  |
| Schuster S J. N Engl J Med 2019;380:45-56. | Non-Randomized, Single Group Assignment | adults | Adult Relapsed  or Refractory Diffuse Large B-Cell Lymphoma | Tisagenlecleucel | NA | NA | phase 2a | 111 | 0 | NA | CRS: grade >3 22% (24/ 111); NSD: grade >3: 12% (13/ 111) | NA |  |
| Locke F L. The lancet oncology, 2019, 20(1): 31-42. | cohort | adults | refractory large B-cell lymphoma | axicabtagene ciloleucel | NA | NA | phase 1/2 | 108 | 4 | Axicabtagene ciloleucel‒unrelated event of pulmonary embolism:(1);Axicabtagene ciloleucel-related events of hemophagocytic lymphohistiocytosis(1) and  cardiac arrest(1) in the context of CRS, and axicabtagene ciloleucel‒unrelated events of intracranial hemorrhage(1) | CRS: grade3,4,5: 11% (12/108) NSD: grade3,4,5: 32% (35/108) | 18d,16d,16d |  |
| Xiuli Wang, Blood. 2016;127(24):2980-2990 | Single Group Assignment | ≥ 18 | relapsed intermediate-grade B-cell non-Hodgkin lymphoma | CD19 CAR–T cells | The first generation and second-generation﻿ | 1 | Phase 1 | 8 | 0 | NA | NA | NA |  |
| Rebecca A. Gardner. Blood. 2017;129(25):3322-3331 | Non-Randomized, Sequential Assignment | >12 months and ＜27 years | relapsed or refractory B-lineage acute lymphoblastic leukemia | CD19 CAR–T cells | NA | NA | 1 | 45 | 0 | NA | CRS: grade 3,4: 22% (10/45); NSD:grade 3,4: 20% (9/45) | NA |  |
| Turtle C. J. J Clin Invest. 2016;126(6)_2123-38. | Single Group Assignment | adults | adults with B cell acute lymphoblastic leukemia after  lymphodepletion chemotherapy. | CD19 CAR–T cells | NA | NA | phase 1 | 30 | 2 | severe CRS and multiorgan failure (1); irreversible neurologic toxicity: (1) | CRS: grade 3: 23% (7/30); NSD: grade 3: 50% (15/30) | 3d;122d |  |
| Kochenderfer J N. J Clin Oncol 33:540-549. | Non-Randomized, Sequential Assignment | adults | advanced B-cell malignancies | CD19 CAR T cells Tisagenlecleucel | NA | NA | phase 1 | 15 | 1 | maybe cardiac arrhythmia | CRS:NA; NSD: grade 3: 69% (9/13) | 16d |  |
| Turtle C J. J Clin Oncol. 2017;10;35 (26) 3010 | Single Group Assignment | adults | chronic lymphocytic leukemia (CLL) who had previously received ibrutinib | CD19 CAR-T cells﻿ | NA | NA | phase I /II | 24 | 1 | CRS and neurotoxicity | CRS: grade 3,4,5 :50% (12/24); NSD: grade3,4:25% (6/24) | NA |  |
| Kevin A. Hay Blood. 2017;130(21):2295-2306 | Single Group Assignment | adults | relapsed and/or refractory CD191 B-cell malignancies | CD19-CAR | The first-generation﻿ | NA | phase I /II | 133 | 6 | complications associated with CRS and/or neurotoxicity: (5); irreversible neurotoxicity: (1) | CRS: grade3,4,5: 12% (16/133); NSD: grade >3, 21% (28/133) | the first 30 days;4month |  |
| Daniel W Lee. The Lancet, 2015, 385(9967): 517-528. | Non-Randomized, Sequential Assignment | aged 1–30 years | relapsed or refractory acute lymphoblastic leukaemia or non-Hodgkin lymphoma﻿ | CD19-CAR | NA | NA | phase 1 | 21 | 0 | NA | CRS: grade 3,4: 29% (6/21); NSD: grade 3,4: 5% (1/21) | NA |  |
| Park J H, M.D.N Engl J Med 2018;378:449-59. | cohort | adults | relapsed B-cell acute lymphoblastic leukemia (ALL) | 19-28z CAR T cells | The second-generation﻿ | NA | phase 1 | 53 | 1 | severe cytokine release syndrome | CRS: grade >3: 26% (14 / 53); NSD: grade >3: 41% (22 / 53) | 5d |  |
| Stephen J. S. N Engl J Med. 2017 Dec 28;377(26)_2545-2554. | Randomized, Parallel Assignment | adults | diffuse large B-cell lymphoma or Follicular lymphoma | CD19-Directed CAR-T (CTL019) | NA | NA | phase 1 | 28 | 1 | Neurotoxicity (1): progression neurologic deterioration | CRS: grade >3: 18% (5/ 28); NSD: grade >3: 11% (3 / 28) | NA |  |
| Michael Kalos. Sci Transl Med. 2011, 3(95): 95ra73-95ra73. | Non-Randomized,Single Group Assignment | adults | advanced, chemotherapy-resistant CLL | CD19-targeted T cells | The second-generation﻿ | 3 | phase 1 | 3 | 0 | NA | NA | NA |  |
| Turtle C J. Sci Transl Med. 2016, 8(355): 355ra116-355ra116. | Single Group Assignment | adults | relapsed and/or refractory B cell non-Hodgkin lymphoma | CD19 CAR-T cells were manufactured  from defined T cell subsets (CD4:CD8=1;1) | NA | NA | phase 1 | 32 | 2 | pontine hemorrhage (1), a fatal gastrointestinal (GI)  hemorrhage (1), | CRS: grade >3: 12.5% (4/ 32); NSD: grade >3: 28% (9/ 32) | NA |  |
| Jiang H. Annals of Hematology. 2019: 1-12. | Single Group Assignment | 0-65 | relapsed and refractory (r/r) B cell acute lymphoblastic leukemia (B-ALL) | CD19-targeted chimeric antigen receptor T cell | NA | NA | phase 1/2 | 53 | 1 | alimentary tract hemorrhage | CRS: grade >3: 24% (19/ 53); NSD: grade >3: (28%)9 / 32 | 21d |  |
|  |  |  |  |  |  |  |  | 890 | 33 |  |  |  |  |
